# UHRF1 mediates cell migration and invasion of gastric cancer

**DOI:** 10.1042/BSR20181065

**Published:** 2018-12-18

**Authors:** Haixia Zhang, Yanli Song, Changqing Yang, Xianzheng Wu

**Affiliations:** Department of Emergency, Tongji Hospital Affiliated to Tongji University, Shanghai 200333, P.R. China

**Keywords:** Gastric cancer, Invasion, Migration, ROS, UHRF1

## Abstract

Gastric cancer (GC) is a common highly aggressive malignant tumor in worldwide. Ubiquitin-like with PHD and ring-finger protein 1 (UHRF1) has a key role in several kinds of cancers development. However, the biology effect of UHRF1 on the tumorigenesis of GC remains unclear. In this research, the role of UHRF1 in the growth, migration, invasion and apoptosis and the underlying mechanisms were investigated in MGC803 and SGC7901 cells. The UHRF1 knockdown MGC803 and SGC7901 cell lines were used to investigate the roles of UHRF1 on GC cell growth, migration, invasion and apoptosis. The growth, migration and invasion rate of UHRF1 knockdown cells was lower than that of the control. Moreover, ROS generation and caspase-3/caspase-9 activities increased in UHRF1 knockdown cells. And mitochondrial membrane potential decreased in UHRF1 knockdown cells. These findings indicated that UHRF1 promoted the growth, migration and invasion of MGC803 and SGC7901 cells and inhibited apoptosis via a ROS-associated pathway.

## Introduction

Gastric cancer (GC) is one of the most leading causes of cancer-related mortality [1–3]. More important, an increase rate of GC has been reported in developing countries, including China [4]. In spite of improvements in diagnostic techniques and treatment, the 5-year relative survival rates are still poor in GC patients at an advanced stage [5,6]. The poor prognosis of GC presents at an advanced stage by its character of invasion and metastasis [7,8]. As invasion and metastasis have a critical function in the GC progression, revealing the mechanisms of the invasion and metastasis of GC and identification of new biomarkers are important for early diagnosis and effective therapeutic strategies.

The epigenetic regulator UHRF1 (ubiquitin-like, containing PHD and RING finger domains 1) has been regarded as a regulator to maintaining DNA methylation [9]. The expression of UHRF1 increases in multiple kinds of cancers, including bladder cancer [10], colorectal cancer [11] and gastric cancer [12]. More and more evidences have revealed that UHRF1 is involved in tumorigenesis. So, UHRF1 may be a potential biomarker for tumor diagnosis and prognosis. UHRF1 increases prostate cancer cells and non-small cell lung cancer cells proliferation [13,14]. However, few investigations are carried out to know whether UHRF1 induced the migration and metastasis of pancreatic cancer cells [15]. The function and mechanism of UHRF1 in tumor cell migration, invasion and carcinogenesis remain largely unknown.

In the present study, we indicated that UHRF1 is overexpressed in GC cell lines and UHRF1 knockdown decreases the proliferation, migration and invasion of GC cells, implied that UHRF1 acts as an oncogene in invasion and migration of GC cells.

## Materials and methods

### Materials

The human GC cell lines MGC803 and SGC7901 cells were purchased from ATCC (American Type Culture Collection, Manassas, VA, U.S.A.). RPMI-1640 culture medium was purchased from Gibco (Grand Island, NY, U.S.A.). Streptomycin, Lipofectamine 2000 and TRIzol were obtained from Invitrogen (Carlsbad, CA, U.S.A.). Antibodies were obtained from Santa Cruz Biotechnology (Santa Cruz, CA, U.S.A.).

### Cell culture

The MGC803 and SGC7901 cells were cultured in RPMI-1640 medium with 10% fetal bovine serum (FBS), 1% penicillin G and streptomycin (Gibco, Grand Island, NY, U.S.A.). All cells were seeded at 37°C in an incubator with 5% CO_2_.

### Plasmid construction and stable short hairpin (sh)RNA transfection

The shRNAs targeting UHRF1 (shUHRF1) and non-targeting control (mock) were obtained by GeneChem Co., Ltd. (Shanghai, China). The transfection of lentiviral vectors with shRNAs against human UHRF1 or control non-target sequence were carried out by Lipofectamine^®^ 2000 transfection reagent (Thermo Fisher Scientific, Inc.) according to the manufacturer’s protocol. The MGC803 and SGC7901 cells were infected for 24 h. Then, the stably transfected cells were treated with puromycin (2 μg/ml) for 48 h.

### Cell proliferation assay

Cell proliferation was determined by the Cell Counting Kit-8 (CCK-8; Dojindo Molecular Technologies, Inc., Kumamoto, Japan) following the instruction. GC cells (5 × 10^3^ cells/well) were seeded into 96-well plates for 24 h. Then, CCK-8 solution (10 μl) was added to each well at 37°C for 1 h. The optical density at 450 nm (OD450) was determined by a microplate reader.

### Annexin V-FITC/PI staining assay

The apoptosis of MGC803 and SGC7901 cells was measured by annexin V-FITC/PI dual labeling assay kit (BioVision, CA, U.S.A.) in accordance with the manufacturer’s protocol. At least 1 × 10^5^ cells of each samples were assessed by a flow cytometer (Becton, Dickinson, CA, U.S.A.).

### Measurement of caspase-3 and caspase-9 activities

The activities of caspase-3 and caspase-9 were detected by a colorimetric kit (Beyotime Institute of Biotechnology, Haimen, China) following the manufacturer’s protocol. The cells (1 × 10^6^ cells/ml) were lysed and the reaction buffer was added, then the caspase-3 or caspase-9 colorimetric substrate (5 μl, DEVD-pNA) was added for 4 h at 37°C. The optical density (OD) was detected by an ELISA reader at 405 nm.

### Wound healing assay

The MGC803 and SGC7901 cells were seeded in six-well plates and reached at least 80% confluence. A 200-μl pipette tip was used to obtain a wound. The cell debris was eliminated by washing with PBS. Images were gained with an Olympus microscope for 0 and 24 h and analyzed with ImageJ.

### Invasion and migration assay

The transwell chamber obtained from Corning Costar (New York, U.S.A.) was used to detect cell invasion and migration. For the cell invasion assay, the chamber was precoated with matrigel (BD Biosciences, U.S.A.) for 1 h at 37°C. Cells (5 × 10^5^ cells) were seeded into the upper chamber, and the lower chamber was added with 1640 medium (500 ml) contained 20% FBS at 37°C for 12 h. The invaded cells were stained with Calcein-AM, and the invaded cells were detected under a fluorescence microscopy (× 100 magnification). For cell migration assay, the chamber was not coated with matrigel.

### Western blot analysis

The cells were collected and lysed. The protein concentration was determined using the BCA method. The protein was separated by sodium dodecyl sulfate-polyacrylamide gel electrophoresis. The protein that was present on 10% SDS-PAGE was placed to nitrocellulose. After blocking non-specific binding, the membranes were incubated overnight at 4°C with different specific primary antibodies in TBST. Membranes were then incubated with secondary antibodies for 1 h at room temperature. The blots were detected by enhanced chemiluminescence reagent and analyzed using ImageJ.

### Statistical analysis

All treatments were performed in three times. Data were analyzed using SPSS 17.0. The statistical significance of differences between groups was determined using *t-*tests. Data are expressed as means ± standard deviations. Results of the Western blot analysis were evaluated using Quantity One software. Differences with *P*-values of less than 0.05 were considered significant.

## Results

### UHRF1 is highly expressed in human GC cell lines

**Figure 1:**
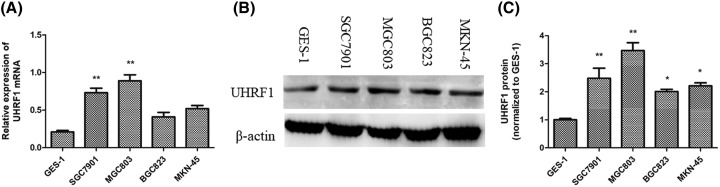
UHRF1 is overexpressed in GC cell lines (**A**) RT-qPCR and (**B** and **C**) Western blotting analyses of UHRF1 expression in five GC cell lines (AGC, SGC7901, MGC803, BGC823 and MKN-45) and GES-1 cells; *P<0.05; **P<0.001.

The expression of UHRF1 was investigated in GC cell lines (SGC7901, MGC803, BGC823 and MKN-45) and the gastric epithelium cell line GES-1 by RT-qPCR and Western blotting assays. The results showed that both mRNA and protein levels of UHRF1 were increased in the GC cell lines (Figure 1A,B, respectively). Among these GC cell lines, the levels of UHRF1 relatively higher in MGC803 and SGC7901. So, MGC803 and SGC7901 cells were used for further investigation.

### Establishment of stably UHRF1 depletion GC cells lines

**Figure 2:**
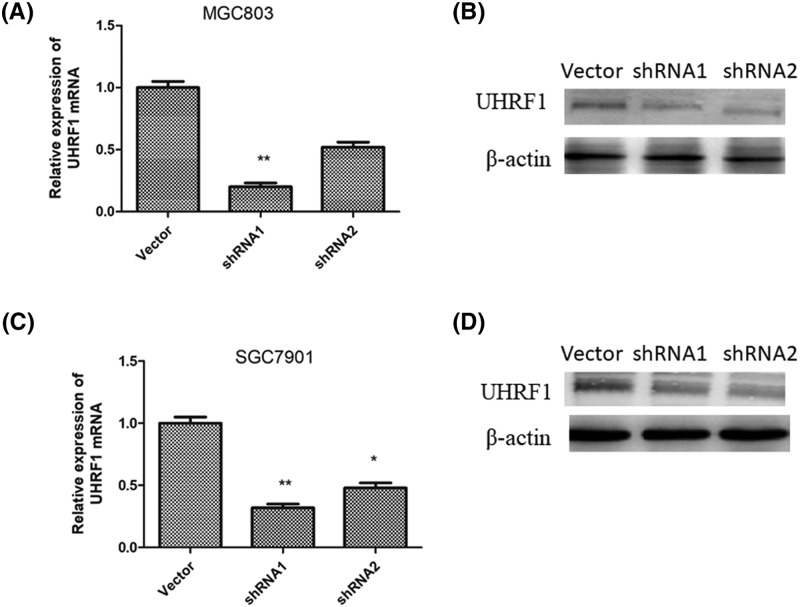
Establishment of stably UHRF1 depletion GC cells lines RT-qPCR and Western blotting analyses of UHRF1 expression in MGC803 (**A** and **B**) and SGC7901 (**C** and **D**) cells infected with UHRF1 shRNA or NC; *P<0.05; **P<0.001.

To investigate the effect of UHRF1 on the GC cells, knockdown of UHRF1 was performed in MGC803 and SGC7901 cells. The knockdown of UHRF1 of MGC803 and SGC7901 was confirmed by RT-qPCR and Western blotting assays. The data showed that UHRF1 is reduced in MGC803 (Figure 2A,B) and SGC7901 (Figure 2C,D). So, knockdown of UHRF1in MGC803 and SGC7901 cells by shRNA1 were used for further investigation.

### Effects of UHRF1 on MGC803 and SGC7901 cells proliferation

**Figure 3:**
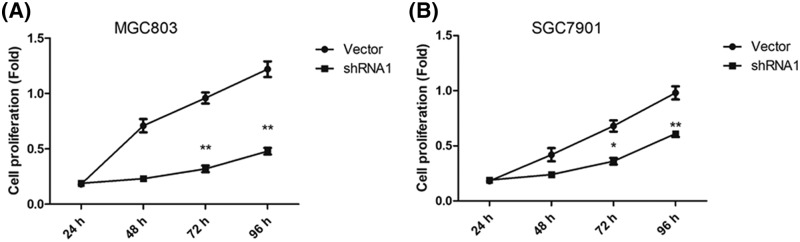
Down-regulation of UHRF1 inhibited GC cell proliferation (**A**) CCK8 assay of MGC803 cells infected with UHRF1 shRNA or NC. (**B**) CCK8 assay of SGC7901 cells infected with UHRF1 shRNA or NC; *P<0.05; **P<0.001.

The CCK-8 assay was used to investigate the effect of UHRF1 on the GC cells proliferative ability. The data demonstrated that the proliferation of UHRF1knockdown cells were decreased compared with that of control group, indicating that knockdown of UHRF1 abrogated the GC cells proliferative ability (*P*<0.05; Figure 3). These findings revealed a promotive role of UHRF1 in the GC cells proliferation.

### Effect of UHRF1 on MGC803 and SGC7901 cells migration

**Figure 4:**
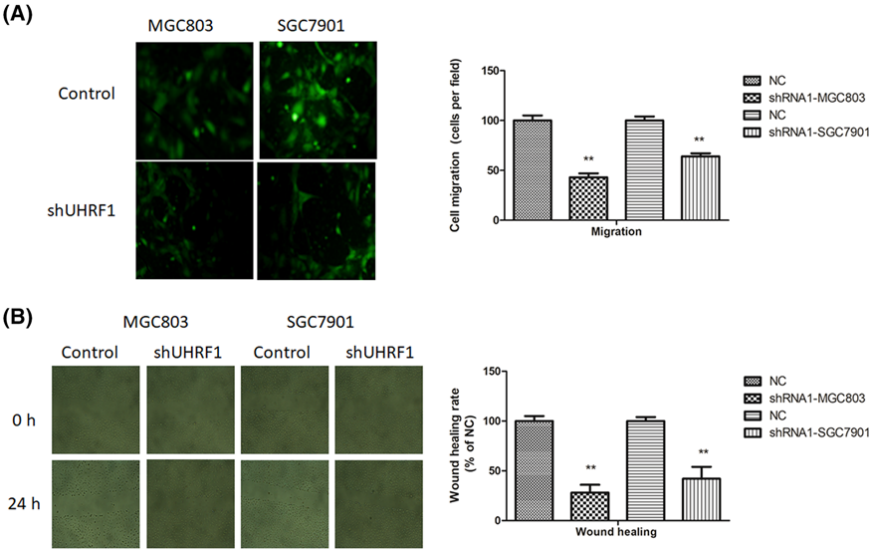
Down-regulation of UHRF1 inhibited GC cell metastasis (**A**) Transwell migration assay of MGC803 and SGC7901 cells infected with UHRF1 shRNA or NC. (**B**) Scratch wound healing assay of MGC803 and SGC7901 cells infected with UHRF1 shRNA or NC. Left panel: representative images. Right panel: quantification of ten randomly selected fields; *P<0.05; **P<0.001.

To investigate the functions of UHRF1 in GC cell migration, transwell assay and the scratch wound healing assay were utilized. Compared with control group, UHRF1 knockdown cells reduced migration ability in the transwell assay (Figure 4A).

Compared with mock control group, UHRF1 knockdown reduced cell migration, as evidenced by a wider scratch area after 24 h (Figure 4B). All of results implied that UHRF1 promotes the migratory capabilities of GC cells.

### Effects of UHRF1 on MGC803 and SGC7901 cells invasion

**Figure 5:**
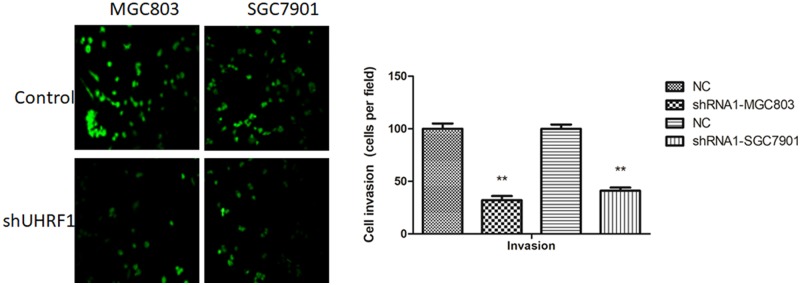
Down-regulation of UHRF1 inhibited GC cell invasion Transwell invasion assay of MGC803 and SGC7901 cells infected with UHRF1 shRNA or NC. **P<0.001.

The invasion ability of GC cells was evaluated by transwell invasion assay. Compared with control group, knockdown of UHRF1 reduced invasion ability of GC cells (Figure 5). These data suggested UHRF1 plays a key role in the induction of GC cells metastasis *in vitro*.

### Effects of UHRF1 on MGC803 and SGC7901 cells apoptosis

**Figure 6:**
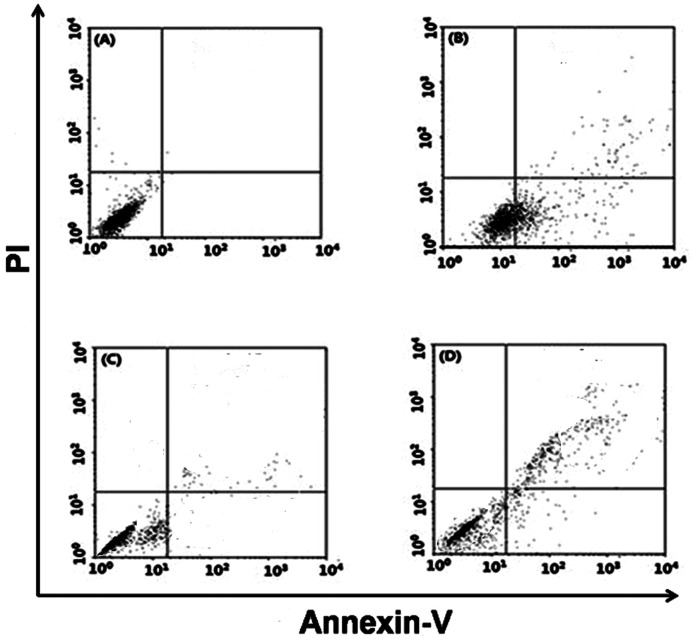
Down-regulation of UHRF1 inducted GC cell apoptosis by FITC/PI assay *P<0.05; **P<0.001

To investigate the functions of UHRF1 in GC cell apoptosis, FITC/PI assay were utilized. Compared with control group, UHRF1 knockdown cells significantly induced apoptosis rates (Figure 6). The early apoptotic cells were enhanced (3.16 ± 0.47% into 21.21 ± 3.16%, 2.47 ± 0.81% into 17.66 ± 1.13%, respectively) and the late apoptotic and necrotic cells were enhanced (0.45 ± 0.01% into 7.09 ± 0.83%, 0.16 ± 0.03% into 2.59 ± 1.13%, respectively, Figure 6).

### UHRF1 reduces ROS generation and mitochondrial membrane potential in MGC803 and SGC7901 cells

**Figure 7:**
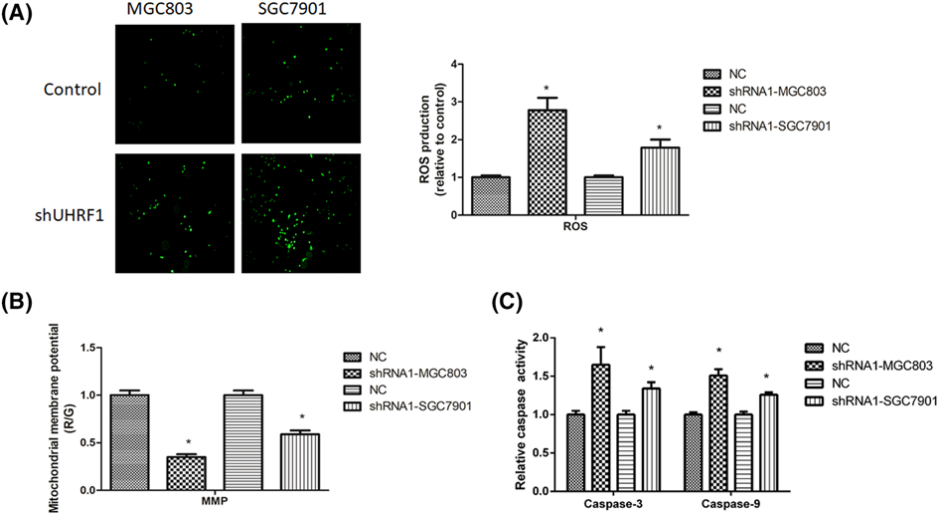
Down-regulation of UHRF1 induced GC cell (**A**) ROS generation, (**B**) MMP, (**C**) Caspase-3 and caspase-9 activity *P<0.05.

As UHRF1 induced the apoptotic rate in GC cells, therefore, we measured the level of ROS. The data showed that UHRF1 knockdown induced ROS generation (Figure 7A).

Induction of ROS generation is related with reduction of MMP. Therefore, we revealed the effect of UHRF1 on MMP. The results showed that UHRF1 knockdown reduces MMP in GC cells (Figure 7B).

The caspase-3 and caspase-9 activities significantly higher in UHRF1 knockdown group than those in the control group (Figure 7C).

## Discussion

The past reports have described a significant association between high UHRF1 expression and poor outcome of bladder cancer [16], breast cancer [17] and pancreatic cancer [18]. So, abnormal expression of UHRF1 is related to carcinogenesis. To the best of our knowledge, there were few previous investigations on the potential role of UHRF1 in GC migration and invasion.

In gallbladder cancer, UHRF1 depletion is associated with cell apoptosis and cell cycle arrest [19]. UHRF1 expression enhances in nasopharyngeal carcinoma tissues and acted a tumor inductor in bladder cancer [20]. In the present study, we have identified that UHRF1 acts as an oncogene in GC. It is identified that down-regulation of UHRF1 was associated with anti-proliferation of GC [21]. We also investigated the migration and invasion effect of UHRF1 on GC. Our results showed that knockdown of UHRF1 decreases the migration and invasion of GC cells. These data implied a promoting role of UHRF1 in the progression of GC.

Oxidative stress is triggered via enhanced intracellular ROS level and reduced antioxidant defense system. The increase of ROS generation would injure intracellular and trigger cell apoptosis [22,23]. The past results revealed that lobaplatin induces the apoptosis of GC cells via ROS apoptotic signal [24]. Moreover, the activities of caspase-3 and caspase-9 have been detected now. In order to study the effect of UHRF1 on oxidative stress, we measured the level of ROS and MMP in GC cells. The data demonstrated that UHRF1 knockdown increases ROS generation and depleted MMP, which ultimately led to elevation of oxidative stress in GC cells. Caspases have a key role in affecting the cell apoptotic process. Once caspases activated, it initiates downstream caspases, promoting to apoptosis. Our data indicated that knockdown of UHRF1 induces GC cells apoptosis via ROS-mitochondrial pathway.

In summary, the data obtained in this investigation exhibited that UHRF1 initiates GC cells proliferation, migration, invasion and inhibits apoptosis. Furthermore, UHRF1 knockdown increased ROS generation and reduced mitochondrial membrane potential. Thus, although our data presented new insights into the roles of UHRF1 in GC, the molecular mechanism of GC request further more investigation. We concluded that UHRF1 may be a new potential target for future therapies of metastatic GC.

## Abbreviations

CCK-8: Cell Counting Kit-8
GC: gastric cancer
UHRF1: ubiquitin-like with PHD and ring-finger protein 1

## References

[1] Zhang L. (2017) The role of tumoral FOXP3 on cell proliferation, migration, and invasion in gastric cancer. Cell. Physiol. Biochem. 42, 1739–1754 10.1159/000479442 28743116

[2] Zhu Y.W. (2016) Knockdown of radixin suppresses gastric cancer metastasis in vitro by up-regulation of E-cadherin via NF-kappaB/Snail pathway. Cell. Physiol. Biochem. 39, 2509–2521 10.1159/000452518 27855404

[3] Ferro A. (2018) Tobacco smoking and gastric cancer: meta-analyses of published data versus pooled analyses of individual participant data (StoP Project). Eur. J. Cancer Prev. 27, 197–204 10.1097/CEJ.0000000000000401 29595756

[4] Cheng X. and Lu Y. (2017) A review of capecitabine-based adjuvant therapy for gastric cancer in the Chinese population. Future Oncol. 14 (8), 771–779 10.2217/fon-2017-0558 29252007

[5] Lisiecki R. (2017) Prognostic significance, diagnosis and treatment in patients with gastric cancer and positive peritoneal washings. A review of the literature. Rep. Pract. Oncol. Radiother. 22, 434–440 10.1016/j.rpor.2017.08.004 28883764 PMC5581864

[6] Shi J. (2017) Efficacy and safety of taxane-based systemic chemotherapy of advanced gastric cancer: A systematic review and meta-analysis. Sci. Rep. 7, 5319 10.1038/s41598-017-05464-0 28706257 PMC5509659

[7] Wang G. (2015) PAK1 regulates RUFY3-mediated gastric cancer cell migration and invasion. Cell Death Dis. 6, e1682 10.1038/cddis.2015.50 25766321 PMC4385928

[8] Gong L. (2018) Overexpression of MYC binding protein promotes invasion and migration in gastric cancer. Oncol. Lett. 15, 5243–5249 29552163 10.3892/ol.2018.7944 PMC5840499

[9] Li T. (2018) Structural and mechanistic insights into UHRF1-mediated DNMT1 activation in the maintenance DNA methylation. Nucleic Acids Res. 46, 3218–3231 10.1093/nar/gky104 29471350 PMC5887372

[10] Zhang Y. (2014) Upregulated UHRF1 promotes bladder cancer cell invasion by epigenetic silencing of KiSS1. PLoS One 9, e104252 10.1371/journal.pone.0104252 25272010 PMC4182677

[11] Wang F. (2012) UHRF1 promotes cell growth and metastasis through repression of p16(ink(4)a) in colorectal cancer. Ann. Surg. Oncol. 19, 2753–2762 10.1245/s10434-011-2194-1 22219067

[12] Hong J.H. (2018) LINE-1 hypomethylation is inversely correlated with UHRF1 overexpression in gastric cancer. Oncol. Lett. 15, 6666–6670 29616129 10.3892/ol.2018.8121 PMC5876460

[13] Wan X. (2016) UHRF1 overexpression is involved in cell proliferation and biochemical recurrence in prostate cancer after radical prostatectomy. J. Exp. Clin. Cancer Res. 35, 34 10.1186/s13046-016-0308-0 26884069 PMC4756440

[14] Chen J. (2018) WDR79 mediates the proliferation of non-small cell lung cancer cells by regulating the stability of UHRF1. J. Cell. Mol. Med. 22, 2856–2864 10.1111/jcmm.13580 29516630 PMC5908104

[15] Cui L. (2015) Up-regulation of UHRF1 by oncogenic Ras promoted the growth, migration, and metastasis of pancreatic cancer cells. Mol. Cell. Biochem. 400, 223–232 10.1007/s11010-014-2279-9 25416862

[16] Yang G.L. (2012) UHRF1 is associated with tumor recurrence in non-muscle-invasive bladder cancer. Med. Oncol. 29, 842–847 10.1007/s12032-011-9983-z 21611839

[17] Yan F. (2011) Inhibition effect of siRNA-downregulated UHRF1 on breast cancer growth. Cancer Biother. Radiopharm. 26, 183–189 10.1089/cbr.2010.0886 21539450

[18] Abu-Alainin W. (2016) UHRF1 regulation of the Keap1-Nrf2 pathway in pancreatic cancer contributes to oncogenesis. J. Pathol. 238, 423–433 10.1002/path.4665 26497117 PMC4738372

[19] Qin Y. (2014) UHRF1 depletion suppresses growth of gallbladder cancer cells through induction of apoptosis and cell cycle arrest. Oncol. Rep. 31, 2635–2643 10.3892/or.2014.3145 24756644

[20] Saidi S. (2017) Overexpression of UHRF1 gene correlates with the major clinicopathological parameters in urinary bladder cancer. Int. Braz J. Urol. 43, 224–229 10.1590/s1677-5538.ibju.2016.0126 28128913 PMC5433360

[21] Zhou L. (2015) UHRF1 promotes proliferation of gastric cancer via mediating tumor suppressor gene hypermethylation. Cancer Biol. Ther. 16, 1241–1251 10.1080/15384047.2015.1056411 26147747 PMC4622020

[22] Mansingh D.P. (2018) [6]-Gingerol-induced cell cycle arrest, reactive oxygen species generation, and disruption of mitochondrial membrane potential are associated with apoptosis in human gastric cancer (AGS) cells. J. Biochem. Mol. Toxicol. e22206 10.1002/jbt.22206 30091159

[23] Liu B.N. (2013) Apoptosis induced by benzyl isothiocyanate in gefitinib-resistant lung cancer cells is associated with Akt/MAPK pathways and generation of reactive oxygen species. Cell Biochem. Biophys. 66, 81–92 10.1007/s12013-012-9456-9 23111983

[24] Li Y. (2016) Lobaplatin induces BGC-823 human gastric carcinoma cell apoptosis via ROS- mitochondrial apoptotic pathway and impairs cell migration and invasion. Biomed. Pharmacother. 83, 1239–1246 10.1016/j.biopha.2016.08.053 27565846

